# Incidence of Potentially Toxic Elements and Perfluoroalkyl Substances Present in Canned Anchovies and Their Impact on Food Safety

**DOI:** 10.3390/foods12051060

**Published:** 2023-03-02

**Authors:** Maria Nobile, Giacomo Mosconi, Luca Maria Chiesa, Sara Panseri, Luigi Danesi, Ermelinda Falletta, Francesco Arioli

**Affiliations:** 1 Department of Veterinary Medicine and Animal Science, University of Milan, Via dell’Universita’ 6, 26900 Lodi, Italy; 2 Department of Chemistry, University of Milan, Via Golgi 19, 20133 Milan, Italy

**Keywords:** fish, anchovy, perfluoroalkyl substances, toxic elements, risk characterization, food safety

## Abstract

Fish plays a key role in a healthy and balanced Italian diet, but it is also subject to the bioaccumulation of different contaminants depending on the geographical or anthropogenic context from which it is derived. In recent years, the European Food Safety Authority (EFSA) has been focusing its attention on consumer toxicological risk, considering emerging contaminants such as perfluoroalkyl substances (PFASs) and potentially toxic elements (PTEs). Regarding fish, anchovies are among the five small pelagic main commercial species in the European Union and the top five fresh species consumed by households in Italy. Considering the lack of data on PFASs and PTEs in this species, our aim was to investigate the mentioned contaminants in salted and canned anchovies collected over 10 months from different fishing areas, even those far apart, to verify possible variations in bioaccumulation and to consider the risk for the consumer. According to our results, the assessed risk was very reassuring also for large consumers. The only concern, related to Ni acute toxicity, also dependent on the different consumers’ sensitivity, was related to only one sample.

## 1. Introduction

Fish constitutes a fundamental component of the Italian diet, representing a relevant source of protein, polyunsaturated fatty acids and micronutrients; however, humans, through the consumption of fish products, are exposed to various contaminants in relation to the quality of the environment from which they are derived [1]. The pollution of marine waters is mainly due to the development of anthropogenic activities that result in the direct or indirect introduction of substances capable of having harmful effects on living organisms—and, consequently, on human health—into the aquatic environment. In particular, it depends on contaminants transported to the sea by rivers and inland watersheds, along which numerous industrial, agro-livestock activities and/or intense urbanization phenomena are present, while a significant contribution is due to the direct sources, in coastal waters, of urban landfills and industrial discharges [2]. The toxicological risk assessment of humans as consumers of fish is part of the broader topic of food safety, which has long been the goal of the European Food Safety Authority (EFSA). In addition to persistent and emerging contaminants, such as perfluoroalkyl substances (PFASs), among others [3], potentially toxic elements (PTEs) may also be of concern to the consumer because of their ability to bioaccumulate along the trophic chain. 

Perfluoroalkyl substances (PFASs), usually, are differentiated as long- or short-chain compounds, but this is reductive as they can be sub-grouped in different ways considering their terminal groups and structures. They have unique chemical–physical properties, such as stability under intense conditions of heat, light and pH and persistence in the environment [4]. They also have surfactant functions, e.g., as water and fat repellents, which is the reason for their wide use in different industry sectors, including food-contact materials, construction and household products, food processing, medical articles, fire-fighting, textiles, electronics, aerospace, automotive, aviation, etc. [5].

Their release and circulation through water and air causes groundwater and drinking water contamination, with subsequent accumulation in animals and humans [6]. Some PFASs are categorized as disruptors of the endocrine hormonal system, toxic for reproduction and the development of a fetus, and are suspected carcinogens. The primary source of PFAS exposure is food, especially fish. PFAS contamination in fish depends on the type of fish, age, geographical area, trophic level, etc. [7,8]. Here, our attention is focused on four PFASs from 2020, to carry out the assessment of the sum of perfluorooctanoic acid (PFOA), perfluorononanoic acid (PFNA), perfluorohexanesulfonic acid (PFHxS) and perfluorooctanesulfonic acid (PFOS), with respect to a tolerable weekly intake (TWI) of 4.4 ng kg^−1^ body weight (bw) per week [9].

On the other hand, the presence of PTEs such as Hg, Cd, As, Pb and others in fishery products represents one of the most serious chemical risks to food safety in the seafood supply chain. Metals are important from two points of view: for their toxicity and for their essentiality. Cu, Fe and Zn, for example, are essential metals that can cause toxic effects at high-concentration intakes. Other metals can be classified as potentially toxic, as well as Pb, Cd and Hg, when ingested for long periods, even at low concentrations [10,11,12].

The progressive increase in sea pollution, the globalization of raw material supply markets and location of processing plants and the growing consumer awareness of food safety issues make this risk a critical factor for the development and competitiveness of the sector. The origin, characteristics, mechanisms and toxic effects of these elements on humans are well known. It should be remembered that in fish products (muscles of fish and crustaceans), the maximum levels (MLs), i.e., maximum allowable residue concentrations of heavy metals, are set by Reg. 1881/2006. In canned and processed products, however, MLs are not set for PTEs.

Regarding fish, anchovies are among the five main small pelagic commercial species in the EU. In 2018, the landings of anchovy in the EU reached a 10-year peak of 135.460 tons, where the trend was led by Spain, followed by Italy, Croatia and Greece. In particular, anchovies are among the top five fresh species (in volume and nominal value) consumed by households in Italy and fall under the quality schemes in the EU seafood sector as Traditional Specialties Guaranteed (TSG), registered up to August 2020 in the EUMOFA report [13]. 

If we consider that small pelagic scombroids (anchovies, herring, mackerel, sardines, etc.) are also used in aquaculture for the production of fishmeal and fish oil, we could consider this process a type of biomagnification phenomenon occurring in aquatic ecosystems, resulting in the contamination of other fish or other animals, and therefore, at the end, of the consumer [1].

In the literature, there are few studies about the detection of PFASs or PTEs in anchovies and, if present, only few samples are considered among different types of fish (Table 1) and never a comprehensive study on both these classes of contaminants, referring also to a single provenance. Liquid chromatography coupled to high-resolution mass spectrometry (HRMS) is a powerful instrumental technique of choice for PFAS investigations, combining high selectivity in the identification through the exact mass of the target and untargeted molecules and high sensitivity in the order of pg g^−1^ added to the high scanning speed with different acquisition mode possibilities. On the basis of the considerations mentioned above, the aim of this study was to detect PFASs and PTEs through a survey on salted and canned anchovies of different types, collected in 10 months from different fishing geographical areas, to verify possible variations in bioaccumulation and to assess the risk for the consumer. In addition, we also considered PFASs, which are a very topical subject, considering the entry into force of the new regulations.

## 2. Materials and Methods

### 2.1. Chemicals and Reagents

Solvents and reagents were obtained from Merck (Darmstadt, Germany). All studied PFASs (perfluorobutanoic acid (PFBA), perfluoropentanoic acid (PFPeA), perfluorohexanoic acid (PFHxA), perfluorobutane sulphonic acid (PFBS), perfluoroheptanoic acid (PFHpA), PFOA, perfluorohexane sulphonate (PFHxS), perfluorononanoic acid (PFNA), perfluorodecanoic acid (PFDA), PFOS, perfluorododecanoic acid (PFDoA), perfluoroundecanoic acid (PFUnDA), perfluorotridecanoic acid (PFTrDA), perfluorotetradecanoic acid (PFTeDA), perfluorohexadecanoic acid (PFHxDA), and perfluorooctadecanoic acid (PFODA)) and the two 13C-labeled internal standards (ISs) perfluoro-(1,2,3,4,5-13C5)nonanoic acid (MPFNA) and perfluoro-(1,2,3,4-13C4)octanesulfonic acid (MPFOS) were purchased from Chemical Research 2000 Srl (Rome, Italy). The purification columns Strata PFAS (WAX/GCB, 200 mg/50 mg/6 mL) were by Phenomenex (Torrance, CA, USA). Hg, Ni, Cd, Cr, As, Pb, Al, Sn and the internal standard Yttrium (Y) of 1000 mg L^−1^ in concentration were from Fisher Scientific (USA), as was HNO_3_ (67–69% superpure) and H_2_O_2_ (30 wt%). 

### 2.2. Sample Collection

A total of 258 sample pools of salted and canned anchovies, each one consisting of at least 10 elemental samples—as described by the COMMISSION REGULATION (EC) No. 333/2007 of 28 March 2007, outlining the methods of sampling and analysis for the official control of the levels of lead, cadmium, mercury, inorganic tin, 3-MCPD and benzo (a) pyrene in foodstuffs comprising PET, aluminum and glass product packages—collected during the period from January 2020 to October 2020 to assess an entire annual production cycle, were distributed according to their different origins: 42 from Tunisia, 71 from the Cantabrian Sea and 143 from the Mediterranean Sea (Croatia). 

### 2.3. Analytical Protocol of PFASs

The protocol has been thoroughly described in our previous work [8]. Briefly, 5 g of homogenized sample was spiked with internal standards at 5 ng g^−1^ in a matrix, followed by the addition of 10 mL of acetonitrile for protein precipitation and extraction. After 1 min of vortexing and 15 min of sonication and centrifugation (2500 *g*, 4 °C, 10 min), the supernatant was dried, resuspended in 5 mL of water and purified by STRATA PFAS cartridges. In particular, 4 mL of 0.3% ammonium hydroxide (NH_4_OH) in MeOH, 4 of mL MeOH and 4 mL of ultrapure water were used during preconditioning, followed by the sample loading. Then, 2 washes with 2 × 4 mL of water were carried out, and 2 × 4 mL of 0.3% NH_4_OH in MeOH was used to elute compounds. After being dried, the extract was resuspended in 200 mL of 20 mM MeOH:ammonium formiate (20:80 *v/v*) and eventually centrifuged in Eppendorf for 2 min if precipitate was present. 

The analysis was performed by an UPLC-HRMS system composed of a Vanquish device (Thermo Fisher Scientific, Waltham, MA, USA) coupled to a Thermo Orbitrap™ Exploris 120 (Thermo Fisher Scientific, Waltham, MA, USA), equipped with a heated electrospray ionization (HESI) source. A Raptor ARC-18 5 µm, 150 × 2.1 mm column (Restek, Bellefonte, PA, USA) was used for the separation. Moreover, a small Megabond WR C18 column, 5 cm, 4.6 mm, i.d. 10 mm, was introduced before the injector to delay eventual PFASs present in the system. Mobile phases A (20 mM aqueous ammonium formate) and B (MeOH) were mixed during the gradient, which started with 20% B, increasing to 95% in 7 min and remaining until the 10th min. After 1 min, the initial conditions were reestablished until the 15th. The flow was set at 0.3 mL min^−1^.

With regard to the detector, the capillary and vaporizer temperatures were set at 330 and 280 °C, respectively, the sheath and auxiliary gas at 35 and 15 arbitrary units (AU) and the electrospray voltage at 3.50 kV in negative mode. The full-scan (FS) acquisition was combined with parallel reaction monitoring (PRM) mode for the confirmatory response based on an inclusion list. The FS worked with a resolution of 60,000 FWHM, a scan range of 150–950 *m/z*, a standard automatic gain control (AGC), an RF lens % of 70 and an automatic maximum injection time. The PRM acquisition operated at 15,000 FWHM, with a standard AGC target, an automatic maximum injection time and scan range mode and an isolation window of 1 *m/z*. Fragmentation of the precursors was optimized with a two-step normalized collision energy (10 and 70 eV). XcaliburTM 4.5 (Thermo Fisher Scientific, Waltham, MA, USA) was the software used.

### 2.4. Analytical Protocol of Potentially Toxic and Essential Elements

These analyses were conducted by inductively coupled plasma optical emission spectroscopy (ICP-OES, Optima 8000, Perkin Elmer, Waltham, MA, USA) detection, which allows quantification at ppb levels for all compounds examined, consistent with the legal limits set for some metals. The method parameters were related to digestion using a microwave digestion system (Mars One, CEM Corporation, Matthews, NC, USA) equipped with TFM closed vessels, followed by metal analyses. For the digestion procedure, 1.2 g of sample was accurately weighed into a TFM vessel. The sample was properly mineralized with 6 mL HNO_3_ (67–69% superpure), 2 mL H_2_O_2_ (30 wt%) and 2 mL ultrapure H_2_O. After acid digestion, the sample was cooled and diluted by ultrapure water (Milli-Q™ system, Millipore, MA, USA) to the final volume of 30 mL. At the end, the sample was subjected to instrumental analysis by the ICP-OES technique.

### 2.5. Validation of PFAS Protocol

Validation of the method was carried out following the SANTE Guidance 11312/2021 [26]. Specificity/selectivity was evaluated by analyzing 20 blank samples, verifying the absence of interferences by the lack of peaks with a signal-to-noise ratio S/N >3 close to the retention times of selected analytes. The matrix-matched calibration curves were constructed by spiking 5 g of blank anchovy sample with the standards for five calibration points (0.05, 1, 3, 5 and 10 ng g^−1^) in duplicate. The limit of quantification (LOQ) of the methods was the lowest spiked level meeting the requirements of recovery within the range of 70–120% and an RSD ≤ 20%, assessed on 6 replicates. Recoveries were calculated at LOQ for all compounds, and thus on 6 replicates, by comparing the peak areas of PFASs spiked before extraction to those spiked after extraction. The matrix effect was also calculated by comparing the peak areas of PFASs spiked after extraction of a blank anchovy sample to the peak areas of a standard solution mix, expressed as a percentage. The intra-day repeatability was evaluated through 6 replicates and expressed as repeatability and within-laboratory reproducibility (%RSD), and the inter-day precision by 6 replicates in 3 different days, using one-way analysis of variance (ANOVA).

### 2.6. Validation of PTE Protocol

For the evaluation of the concentrations of PTEs in the samples, the instrument was calibrated with standard solutions of concentrations between 14 and 200 μg kg^−1^, which were properly prepared from available stock solutions. Yttrium was used as an internal standard and high-purity argon was used as an inert gas. Method validation was carried out by evaluating each metal’s limit of detection (LOD), limit of quantification (LOQ) and precision (% RSD) through six replicates. Determination of metal concentrations was carried out in triplicate per sample. 

The metals’ recovery from the matrix was evaluated by the use of a certified reference material (ERM-CE278k mussel tissue, Joint Research Centre Institute for Reference Materials and Measurements, EU) as reported in Table 2, and the metal concentration was corrected for the results.

### 2.7. Statistical Analysis

Statistical analyses were carried out using Graphpad Instat 3 software (Graphpad Instat Software, San Diego, CA, USA). Due to the extremely varying numbers of fishing areas, a statistical comparison between the various provenances could not be carried out with a reliable result. Therefore, the Kolmogorov–Smirnov test was only performed on the entire number of samples to test whether the distribution of the concentration values of the different elements was normal or not (*p* < 0.05). None of the compounds and elements analyzed were found to be normally distributed. Instead, the mean, median and maximum concentration values of the different elements were calculated. 

## 3. Results and Discussion

### 3.1. Validation Performance of PFAS Method

The PFAS protocol validation parameters are reported in Table 3, verifying all the requirements set by SANTE 11312/2021 [26]. Briefly, the method had high selectivity with an S/N >3, where analytes were present, starting from the LOQ level, and high specificity, with the absence of interference close to the retention time of the detected PFASs. The recoveries ranged between 70 and 120%, revealing the good efficiency of the analytical protocol. Precision with RSD lower than 20% was in accordance with the tolerance range indicated. The LOQs in the range from 0.050 to 0.10 ng g^−1^ demonstrated good method sensitivity on this complex matrix, and they perfectly complied with the indicative levels recommended in fish by the new European Recommendations [27]. Matrix calibration curves revealed a good fit over the five calibration points, with *R*^2^ > 0.99 for all PFASs. The matrix effect showed a lower influence (<20%) with a percentage variation from 89 to 106%.

### 3.2. Validation Performance of PTE Method

The PTE protocol validation parameters are reported in Table 4, containing the limit of detection (LOD) and the limit of quantification (LOQ), along with the precision, expressed as % RSD.

### 3.3. Risk Characterization

The average Italian consumption of processed anchovies, both in oil and in salt, was first assessed. We estimated the Italian consumers based on the Italian National Institute of Statistics [28] data on the population updated for June 2021, subtracting roughly 10%, a value corresponding to the subgroup of the population represented by children aged between 0 and 10 years. The reason for their exclusion lies in the presumable difficulty of consumption presented by such individuals for a food with a particular taste, but also in the need to decrease the consumer population to make the subsequent data more precautionary.

Following this, risk characterization was carried out considering a population of 53,961,715 inhabitants, and the “EUMOFA case study (European market observatory for fisheries and aquaculture products): anchovy transformed in Italy, February 2018” was taken into consideration.

In this case study, which reports the data of the Italian market in 2015, the anchovies consumed in Italy were calculated at 43,830 tons of live weight per year, of which 38% are processed, corresponding to 16,484 tons of live weight per year. The case study reports that starting from 2.25 kg live weights of anchovies, 0.57 kg of clean fillet is produced, i.e., only 25.3% of the initial weight is transformed into the final product.

If the final yield is 25.3% [29], the apparent consumption of transformed anchovies is 4170 tons per year, with a per capita consumption figure of 0.08 kg per person per year, corresponding to 0.22 g per person per day.

Subsequently, the Estimated Daily Intake (EDI) of the PTEs and PFASs was calculated as follows:

EDI = C × DC/BW,(1)

where C is the mean concentration (considering that the mean was always equal to or higher than the median and therefore precautionarily considered), DC is the daily fish consumption per capita in Italy and BW is the consumer bodyweight, considered equal to 70 kg. We also considered the 95th percentile estimated seafood consumption, which was calculated as 2.9 times that of the median or mean consumers [30].

We also calculated the Target Hazard Quotient (THQ), which is the ratio between the exposure and the health-based guidance value (HBGV) indicated by EFSA (2020) for each element/compound and each end-point, eventually recalculated on a daily basis:

THQ = EDI/HBGV.(2)

For a very conservative approach, the highest concentrations were accounted for. 

The Hazard Index (HI) [31,32] was therefore calculated as

(3) $$HI=\overset{n}{\underset{i=8}{\sum }}THQ$$

Finally, the *HI*s for the EDI via preserved anchovies of the 95th percentile fish consumers were calculated.

The difference in the number of samples from the different fishing areas did not allow a statistical comparison with a reliable result. Moreover, the distribution of the elements was not Gaussian. We therefore calculated the values of the average, median and maximal concentrations of the analyzed elements, as reported in Table 5.

With an analogous argument, the data on the detected PPFAS are reported in Table 6.

When the analyte was not quantifiable, the value corresponding to half the LOQ was used to calculate the average concentration.

The toxicity and the Health-Based Guidance Values (HBGVs), relating to the elements and PFASs analyzed in anchovies, are shown in Table 7.

Table 8 shows the data relating to the estimated daily intakes of elements and PFASs. Note that arsenic is considered only for a tenth of its value found in the analysis. This is because only inorganic arsenic (iAs), 10% of total arsenic, is considered toxic. It must be highlighted that only PFOS and PFOA were detected in the samples. Therefore, the group TWI stated by EFSA for four PFASs [9] refers to the sum of the relieved two of the four PFASs.

Only Hg and Sn exceeded 1% of the safe exposure and only considered the maximum values detected. It should be highlighted that the value of the four most toxic organic components of tin was considered as the toxicity value, and, as a further precautionary approach, the tin detected in the analyzed anchovies was represented only by these four compounds. Furthermore, if only the average and median values measured are taken into consideration, an even more favorable situation is observed, represented by approximately 0.5% of the safe exposure for tin and mercury, and even less than 0.5% of the safe exposure for all other elements.

The sum of PFOA and PFOS, the two found PFASs of the four considered by EFSA for the evaluation of the TWI, represent 0.06 and 0.5 percent of the TWI for the mean and the highest concentrations detected. It must moreover be noticed that the two highest concentrations of PFOS and PFOA did not belong to the same sample, thus making the calculation safer.

Additionally, calculating the THQs as above described and considering the highest concentrations detected, the HIs were always lower than 0.02, thus indicating the absence of concern deriving from the consumption of the preserved anchovies (Table 9). As the cancer risk regards only As, only the non-carcinogenic effects were considered.

Following the considerations, the outcome of the oral exposure risk characterization of the researched elements and PFASs shows that, from a chronic toxicity point of view, the consumption of preserved anchovies does not constitute a cause for concern in relation to the sampling data from January to October 2020.

The EDI for large consumers also shows that this population subclass is not a matter of concern as regards PTEs and PFASs in preserved anchovies (Table 10). 

Finally, the acute toxicity of Ni, consisting of contact dermatitis, must be considered. However, due to the peculiarity of the preserved anchovies, it is not easy to define consumption in terms of fish meal. Unlike fresh fish, preserved anchovies are usually an ingredient—for example, for pizza or some pasta sauces. We accounted for individuals of 70, 55 and 40 kg of body weight as representative of a man of average build and height, a woman of medium build and height and a woman of slender and not particularly tall height. 

A meal of 15 g is the usual serving size of anchovies: the aforementioned individuals could eat 18.4 g, 14.5 g and 10.5 g, respectively, of the sample with the highest concentration (0.418 μg kg^−1^) without incurring allergic dermatitis. Therefore, due to the acute reference value of 1.1 μg kg^−1^ bw Ni with an Moe value of 10 that accounts for the different individual susceptibilities, the portion should be reduced, but only for hypothetical consumers of 55 kg and 40 kg in weight. Considering the second highest value (0.201 μg kg^−1^), the consumption could double to 38.3, 30.1 and 21.9, considerably decreasing the risk of systemic contact dermatitis.

Based on our results, only the sample with the highest content of Ni could cause an allergic reaction in Ni-sensitive, low-weight individuals, thus representing a moderate concern. 

## 4. Conclusions

Considering the lack of data on PFASs and PTEs in anchovies, the aim of the present work was to investigate the mentioned contaminants in salted and canned anchovies collected from different geographical areas in different months of the year to verify possible variations in bioaccumulation. For this purpose, two analytical methods were developed, one for PFASs and one for PTEs. The PFAS method showed high selectivity/specificity, good recovery from 70 and 120%, good sensitivity with LOQs in the range of 0.050 to 0.10 ng g^−1^, high precision (RSD < 20%) and a lower matrix effect (<20%). Additionally, the PTE method showed good sensitivity (LOD from 0.400 to 3.60 ng g^−1^ and LOQ from 1.20 to 12.1 ng g^−1^) and precision (RSD < 25%). 

A total of 258 sample pools of salted and canned anchovies were analyzed. Regarding PFASs, only PFBA, PFOS and PFOA were detected (from <LOQ to 8.40 ng g^−1^); instead, Hg, Cd, Pb, Cr, iAs, Sn, Al and Ni were the elements detected in the anchovies, with concentrations from 90.00 to 4940 ng g^−1^.

According to our results, a risk characterization of the exposure to elements and PFASs was carried out for the Italian consumer, thus permitting us to comprehend the average European consumer. The very low hazard indexes for cumulative toxicities for consumers of large amounts were very reassuring. The only concern derived from Ni acute toxicity, which, also accounting for different individual sensitivities, related to only one sample.

## Figures and Tables

**Table 1:** State of the art on detection of PFASs and potentially toxic elements in fresh and canned anchovies.

**PFASs**						
**Reference**	**Analytes**	**Matrix**	**Extraction** **Technique**	**Instrumental Analysis**	**LOD–LOQ** **ng g^−1^**	**Application** **Range Conc.** **ng g^−1^**
[14]	5 PFCAs; 3 PFSAs	Different fish including 5 anchovies	Alkaline digestion, SPE	LC-MS/MS	0.0030–0.050	<LOQ–0.80
[15]	13 PFCAs; 4 PFSAs	Different foods including anchovies and sardines	Basic methanol extraction, acidification, SPE WAX	LC-MS/MS	0.00090–0.46	0.0090–9.3
[16]	PFOS	Eggs, 5 sardines, 5 anchovies	Acetonitrile extraction, incubation, purification by activated carbon and glacial acetic acid	LC-MS/MS	0.023	0.54–1.5
[17]	9 PFCAs; 3 PFSAs; PFOSA	Different fish including anchovies	Basic methanol extraction, SPE WAX	LC-MS/MS	0.48–10	0.51–15
[18]	7 PFCAs; 3 PFSAs; 3 PFOSA, 2 diPAP, 3 PFPiA, 2 FTCA, 1 FTUCA	Water, sediments and small fish including 15 anchovies	Alkaline digestion, methanol extraction, Pesti-Carb cartridges clean up	LC-MS/MS	0. 00020–0.056	0.011–0.47
[19]	5 PFCAs; 3 PFSAs; PFOSA	Different fish including 8 sardines	Methanol extraction, concentration, treatment with aqueous KOH, SPE WAX	LC-MS/MS	1.0	0.010–3.6
**Heavy metals**						
**Reference**	**Analytes**	**Matrix**	**Extraction** **Technique**	**Instrumental Analysis**	**LOD–LOQ** **ng g^−1^**	**Application** **Range Conc.** **ng g^−1^**
[10]	Fe, Zn, Cu, Cd, Sn, Hg and Pb	Canned anchovies and canned rainbow trout	Digestion with concentrated (65%) nitric acid (HNO_3_) 30% hydrogen peroxide (H_2_O_2_), microwave and washing	ICP–MS	/	1.0–5.1 × 10^4^
[20]	Pb, As, Cd, Zn, Cu	Canned seafood products	Homogenization, drying, digestion with (HCl:HNO_3_ = 1:1), evaporation	Atomic absorption spectrometer	/	27 × 10–7.1 × 10^4^
[21]	Li, Na, Mg, P, Ca, V, Mn, Fe, Co, Ni, Cu, Zn, Ga, As, Se, Rb, Sr, Mo, Pd, Cd, Cs, Ba, Hg, Tl, Pb, U	Sardine and anchovy from 6 Greek coastal areas	Freeze-drying, homogenization, microwave-assisted acid digestion	ICP–MS	2.0–14 x10^4^	40–18 × 10^4^
[22]	As, Cd, Co, Cr, Cu, Mn, Mo, Ni, P, Pb, V, Zn, Ca, K, Na, Mg, S and Sr	Indian anchovy	Digestion with 65% nitric acid	ICP-OES	1.0–4.9 × 10^5^	40–75 × 10^5^
[23]	Al, Zn, Mn, Co, Cr, Cu, Fe, Ni, Cd, Pb, Se, As and Hg	Anchovy of Black Sea	Homogenization, drying, digestion with nitric acid and hydrochloric acid, dilution, filtration	ICP-MS	0.10–29	3.0–14 × 10^2^
[24]	Cr, Mn, Fe, Co, Ni, Cu, Zn, As, Cd, Pb	Fresh and salt-dried anchovy from Kuala Terengganu	Drying, digestion with deionized water–nitric acid (49:1, *v/v*)	ICP-MS	4.0	4.0–65 × 10^4^
[25]	Hg, Cd and Pb	Salted anchovies	Digestion with HNO_3_-HClO_4_ (8:3) for Cd and Pb and with H_2_SO_4_-HNO_3_ (1:1) for Hg	Atomic absorption spectroscopy	5.0–10	40–50 × 10

/: not reported in the article; PFCAs: perfluoroalkyl carboxylic acids; PFSAs: perfluoroalkyl sulfonic acids; PFOSA: perfluorooctane sulfonamide; diPAP: fluorotelomer phosphate diester; PFPiA: perfluorophosphinate; FTCA: perfluoroakyl saturated carboxylates; FTUCA: perfluoroakyl unsaturated carboxylates; LC-MS/MS: Liquid Chromatography tandem mass spectrometry; ICP–MS: Inductively coupled plasma mass spectrometry; ICP-OES: Inductively coupled plasma - optical emission spectrometry.

**Table 2:** Metal concentration reported in the certificate of the reference material, metal concentration in the certified reference materials by ICP-OES and percentage of metal recovery from the reference material.

Metal	Certificate of Analysis of the Reference Material (ng g^−1^)	From ICP-OES Analysis (ng g^−1^)	Recovery (%)
As	6700.00	6740.00	101
Cd	340.000	320.000	94
Pb	2180.00	1990.00	91
Ni	690.000	640.000	93
Cr	730.000	770.000	105
Al	Not reported	136,900	-
Hg	71.0000	160.000	125
Sn	Not reported	270.000	-

-: Not calculated.

**Table 3:** PFAS list with their formula, parent exact mass and validation performance.

Compound	Formula	Parent Exact Mass [*m/z*]	Observed Parent Mass [*m/z*]	Main Fragment ion [*m/z*]	LOQ (ng g^−1^)	Recovery %	Intra-Day % RSD	Inter-Day % RSD
PFBA	C_4_HF_7_O_2_	212.97920	212.97929	168.98955	0.050	115	7	18
PFPeA	C_5_HF_9_O_2_	262.97601	262.97609	218.98612	0.050	114	11	11
PFBS	C_4_F_9_HO_3_S	298.94299	298.94290	98.95434	0.050	104	11	12
PFHxA	C_6_HF_11_O_2_	312.97281	312.97279	268.98352	0.050	117	7	10
PFHpA	C_7_HF_13_O_2_	362.96962	362.96959	318.97977	0.050	113	6	10
PFHxS	C_6_F_13_HO_3_S	398.9366	398.93650	79.95743	0.050	90	6	9
PFOA	C_8_HF_15_O_2_	412.96643	412.96638	368.97698	0.050	113	6	9
PFNA	C_9_HF_17_O_2_	462.96323	462.96318	418.97384	0.050	92	10	14
PFOS	C_8_F_17_HO_3_S	498.93022	498.93024	79.95741	0.050	94	12	15
PFDA	C_10_HF_19_O_2_	512.96004	512.96008	468.96990	0.050	86	11	16
PFUnDA	C_11_HF_21_O_2_	562.95684	562.95679	518.96770	0.050	85	8	13
PFDS	C_10_F_21_HO_3_S	598.92383	598.92377	79.95743	0.050	94	9	13
PFDoA	C_12_HF_23_O_2_	612.95365	612.95360	568.96405	0.050	74	9	13
PFTrDA	C_13_HF_25_O_2_	662.95046	662.95041	618.96097	0.10	80	8	15
PFTeDA	C_14_HF_27_O_2_	712.94726	712.94719	668.95808	0.10	88	15	20
PFHxDA	C_16_HF_31_O_2_	812.94088	812.94078	768.94913	0.10	88	16	20
PFODA	C_18_HF_35_O_2_	912.93449	912.93440	868.94544	0.10	87	17	20

All compounds were detected in the deprotonated form.

**Table 4:** Limit of detection (LOD), limit of quantification (LOQ) and precision (% RSD) of the investigated PTEs.

Element	LOD (ng g^−1^)	LOQ (ng g^−1^)	RSD %
Hg	0.70	2.3	9
Cd	0.60	1.9	3
Pb	2.6	8.6	22
Cr	0.60	2.1	3
As	3.6	12	22
Sn	2.0	6.7	25
Al	0.40	1.2	5
Ni	1.3	4.2	8

**Table 5:** Mean, median and maximum concentrations of elements detected in the anchovies.

Concentration (ng g^−1^)	Hg	Cd	Pb	Cr	iAs	Sn	Al	Ni
**Positives %**	71	100	99	100	100	100	100	100
**Mean**	290.0	60.00	170.0	90.00	230.0	200.0	1880	50.00
**Median**	280.0	50.00	170.0	80.00	210.0	200.0	1720	50.00
**Maximum**	650.0	90.00	750.0	710.0	510.0	670.0	4940	420.0

**Table 6:** Mean, median and maximum concentrations of PFASs detected in the anchovies.

Concentration (ng g^−1^).	PFBA	PFOA	PFOS
**Positives %**	100	1	83
**Mean**	2.08	<LOQ	0.0860
**Median**	1.76	0	<LOQ
**Maximum**	8.40	<LOQ	1.15

**Table 7:** Potential toxicity and Health-Based Guidance Values of searched elements and PFAS.

Element/ PFAS	Potential Toxicity	Health-Based Guidance Value
Mercury	Nervous system dysfunction such as tremors, irritability, memory problems, impaired vision and hearing. Exposure of mothers could lead to the birth of babies with permanent dysfunction of the nervous system.	TWI ^a^ = 1.3 µg kg^−1^ bw per week of methylmercury, expressed as mercury for neurodevelopmental outcomes after prenatal exposure [33]
Cadmium	Kidney and respiratory diseases	TWI ^a^ = 2.5 µg kg^−1^ bw per week [34] for tubular damage
Lead	Severe brain and kidney damage, possible miscarriage	BMDL_10_^b^ Neurodevelopmental toxicity: 0.50 µg kg^−1^ bw per day Blood pressure: 1.5 µg kg^−1^ bw per day kidney: 0.63 µg kg^−1^ bw per day [35]
Chromium	Respiratory problems, cough, asthma and allergic reactions. Chronic exposure could cause liver and kidney cancer, in particular, linked to Cr (VI), which, however, is rare to find in food due to its reduction to Cr (III).	TDI ^c^ = 300 µg kg^−1^ bw per day of Cr (III)) [36] on reproductive and developmental toxicity
Arsenic	In its organic form, it has negligible toxicity due to the fast excretion kinetics. In the form of inorganic arsenic, less than 10% of the total arsenic in fish is linked to skin, lung and bladder cancer.	BMDL_01_^d^ for skin lesions and lung, bladder and skin cancers, ranges 0.30 and 8.0 µg kg^−1^ bw per day [37,38]
Tin	Inorganic tin interferes with the metabolism of zinc, copper and iron, with the synthesis and catabolism of the heme group.	Metallic tin and inorganic tin compounds are relatively nontoxic [39]. TDI ^c^ of 0.10 µg kg^−1^ bw per day for immunotoxic effects of tributyltin, dibutyltin, triphenyltin and di-n-octyltin. This very precautionary value for organic tin (the last two used as additives in PVC and in materials in contact with food) is considered [40].
Aluminium	For professional exposure only, the target organs are the lungs and bones. The toxicity on the central nervous system (CNS) includes dementia in dialyzed patients (due to aluminum entering the circulation with dialysis) or oral exposure to Al hydroxide administered to patients and Parkinson’s disease; however, it should be emphasized that in the two syndromes, the serum or cerebral levels of aluminum could be an effect of the syndrome and not the cause.	TWI ^a^ = 1.0 mg kg^−1^ bw per week for effects on the developing nervous system [41]
Nickel	Long-term toxicity: cancerogenic, immunotoxic, hepatotoxic, neurotoxic and nephrotoxic only through inhalation. Long-term exposure could cause reproductive diseases. Acute toxicity: allergic and eczematous reactions in sensitive individuals.	TDI ^c^ = 2.8 µg kg^−1^ bw per day reproductive and developmental toxicity BMDL_10_^b^ = 1.1 µg kg bw with a margin of exposure (MOE) equal to or greater than 10, accounting for the variability of the response in sensitized individuals [42]
PFAS	Toxicity on immune system, on reproduction and development	TWI = 4.4 ng kg^−1^ bw per week for the sum of PFOA, PFOS, PFHxS and PFNA for the decrease in immune response to vaccination individuals exposed even during the mother’s pregnancy.

^a^ TWI = tolerable weekly intake; ^b^ BMDL_10_ = benchmark dose—lower bound 10%; ^c^ TDI = tolerable daily intake; ^d^ BMDL_01_ = benchmark dose—lower bound 0.1%.

**Table 8:** Data relating to the characterization of the risk from oral exposure to the studied elements through the consumption of preserved anchovies (HBGVs and EDIs of elements expressed as µg kg^−1^ bw per day; HBGV and EDI of PFAS as ng kg^−1^ bw per day).

	Hg	Cd	Pb	Cr	iAs	Sn	Al	Ni	PFOA + PFOS
HBGV ^a^	0.180	0.380	0.500	300	0.300	0.100	143	2.80	0.630
EDI_mean_	0.000910	0.000190	0.000530	0.000280	0.000720	0.000630	0.00590	0.000160	0.000350
EDI_Maximum_	0.00200	0.000280	0.00240	0.00220	0.00160	0.00210	0.0160	0.00130	0.00370

^a^ The HBGVs are reported on a daily basis even when they are stated by EFSA on a weekly basis.

**Table 9:** Hypothetical target hazard quotients and hazard indexes for the estimated daily exposure (EDI) through preserved anchovies at the highest concentrations detected. All the Health-Based Guidance Values (HBGVs) are by EFSA. TWIs are recalculated and expressed on a daily basis. As is reported as inorganic arsenic and only Cr (III) is considered. EDI and HBGV are expressed as μg kg^−1^ day^−1^ for elements and as ng kg^−1^ day^−1^ for PFASs.

HBGV	Element	EDI	Target Hazard Quotient (HBGV Expressed on a Daily Basis)					
			Neurodevelopment	Kidney	Blood Pressure	Reproduction/Development	Skin	Immune System
TWI	Hg	0.00200	0.0100 (0.180)					
TWI	Cd	0.000290		0.00080 (0.38)				
BMDL_10_	Pb	0.00230	0.00500 (0.500)	0.00400 (0.630)	0.00200 (1.50)			
TDI	Cr	0.00230				0.0000800 (300)		
BMDL_10_	iAs	0.00160					0.0050 (0.3)	
TDI	Sn	0.00210						0.020 (0.1)
TWI	Al	0.0160	0.000100 (142)					
TDI	Ni	0.00130				0.000500 (2.80)		
TWI	PFOS + PFOA							0.000600 (0.630)
Hazard Index			0.0150	0.00480	0.00200	0.000580	0.00500	0.0210

**Table 10:** Hazard indexes for the estimated daily exposure to the studied elements via preserved anchovies of the 95th percentile fish consumers, accounting for the highest concentrations detected.

Hazard Index 95th Percentile Consumers					
Neurodevelopment Hg + Pb + Al	Kidney Cd + Pb	Blood Pressure Pb	Reproduction/Development Cr + Ni	Skin As	Immune System Sn + PFOS + PFOA
0.060	0.014	0.0060	0.0020	0.015	0.060

## Data Availability

The data are available from the corresponding author.

## References

[1] Mantovani A., Ferrari D., Frazzoli C. (2015). Sustainability, security and safety in the feed-to-fish chain: Focus on toxic contamination. Int. J. Nutr. Food Sci..

[2] Chiesa L.M., Nobile M., Ceriani F., Malandra R., Arioli F., Panseri S. (2019). Risk characterisation from the presence of environmental contaminants and antibiotic residues in wild and farmed salmon from different FAO zones. Food Addit. Contam. Part A.

[3] Chiesa L.M., Labella G.F., Panseri S., Pavlovic R., Bonacci S., Arioli F. (2016). Distribution of persistent organic pollutants (POPs) in wild Bluefin tuna (Thunnus thynnus) from different FAO capture zones. Chemosphere.

[4] Christensen K.Y., Raymond M., Blackowicz M., Liu Y., Thompson B.A., Anderson H.A., Turyk M. (2017). Perfluoroalkyl substances and fish consumption. Environ. Res..

[5] European Commission- DG Environment (2020). The Use of PFAS and Fluorine-Free Alternatives in Textiles, Upholstery, Carpets, Leather and Apparel. https://echa.europa.eu/documents/10162/17233/pfas_in_textiles_final_report_en.pdf/0a3b1c60-3427-5327-4a19-4d98ee06f041?t=1619607351696.

[6] EFSA (2008). Perfluorooctane sulfonate (PFOS), perfluorooctanoic acid (PFOA) and their salts. Scientific Opinion of the Panel on Contaminants in the Food chain. EFSA J..

[7] EPA (2009). State of the Great Lakes 2009. Prepared by Environment Canada and United States Environmental Protection Agency. http://binational.net/wp-content/uploads/2014/11/En161-3-1-2009E.pdf.

[8] Chiesa L.M., Pavlovic R., Arioli F., Nobile M., Di Cesare F., Mosconi G., Falletta E., Malandra R., Panseri S. (2022). Presence of perfluoroalkyl substances in Mediterranean Sea and North Italian Lake fish addressed to Italian consumer. Int. J. Food Sci. Technol..

[9] EFSA (2020). Scientific Opinion on the risk to human health related to the presence of perfluoroalkyl substances in food. EFSA J..

[10] Mol S. (2011). Determination of trace metals in canned anchovies and canned rainbow trouts. Food Chem. Toxicol..

[11] Mohamadi S., Mahmudiono T., Zienali T., Sadighara P., Omidi B., Limam I., Fakhri Y. (2022). Probabilistic health risk assessment of heavy metals (Cd, Pb, and As) in Cocoa powder (Theobroma cacao) in Tehran, Iran market. Int. J. Environ. Res. Public Health.

[12] Kruszewski B., Obiedziński M.W. (2018). Multivariate analysis of essential elements in raw cocoa and processed chocolate mass materials from three different manufacturers. LWT.

[13] EUMOFA European Market Observatory For Fisheries And Aquaculture Products. The EU Fish Market 2020 Edition. https://www.eumofa.eu/documents/20178/415635/EN_The+EU+fish+market_2020.pdf.

[14] Llorca M., Farré M., Picó Y., Barceló D. (2009). Development and validation of a pressurized liquid extraction liquid chromatography–tandem mass spectrometry method for perfluorinated compounds determination in fish. J. Chromatogr. A.

[15] Domingo J.L., Jogsten I.E., Eriksson U., Martorell I., Perelló G., Nadal M., van Bavel B. (2012). Human dietary exposure to perfluoroalkyl substances in Catalonia, Spain. Temporal trend. Food Chem..

[16] Bertolero A., Vicente J., Meyer J., Lacorte S. (2015). Accumulation and maternal transfer of perfluorooctane sulphonic acid in yellow-legged (Larus michahellis) and Audouin’s gull (Larus audouinii) from the Ebro Delta Natural Park. Environ. Res..

[17] Sedlak M.D., Benskin J.P., Wong A., Grace R., Greig D.J. (2017). Per-and polyfluoroalkyl substances (PFASs) in San Francisco Bay wildlife: Temporal trends, exposure pathways, and notable presence of precursor compounds. Chemosphere.

[18] Chen M., Wang Q., Shan G., Zhu L., Yang L., Liu M. (2018). Occurrence, partitioning and bioaccumulation of emerging and legacy per-and polyfluoroalkyl substances in Taihu Lake, China. Sci. Total Environ..

[19] Fernandes A.R., Mortimer D., Holmes M., Rose M., Zhihua L., Huang X., Smith F., Panton S., Marshall L. (2018). Occurrence and spatial distribution of chemical contaminants in edible fish species collected from UK and proximate marine waters. Environ. Int..

[20] Ismail S., Azhari S., Ngah C.W.Z.C.W. (2018). Determination of selected toxic metal (As, Cd, Pb) and essential (Zn, Cu) elements in local canned seafood products. AIP Conf. Proc..

[21] Sofoulaki K., Kalantzi I., Machias A., Pergantis S.A., Tsapakis M. (2019). Metals in sardine and anchovy from Greek coastal areas: Public health risk and nutritional benefits assessment. Food Chem. Toxicol..

[22] Alizada N., Malik S., Muzaffar S.B. (2020). Bioaccumulation of heavy metals in tissues of Indian anchovy (Stolephorus indicus) from the UAE coast, Arabian Gulf. Mar. Pollut. Bull..

[23] Karsli B. (2021). Determination of metal content in anchovy (Engraulis encrasicolus) from Turkey, Georgia and Abkhazia coasts of the Black Sea: Evaluation of potential risks associated with human consumption. Mar. Pollut. Bull..

[24] Nurulnadia M.Y., Nik-Nurasyikin N.M.A., Ling K.H., Zahid B.M., Adiana G., Nurlemsha B.I. (2021). Metal concentrations in fresh and salt-dried anchovy, Encrasicholina devisi, and estimation of target hazard quotient for consumers in Kuala Terengganu. Reg. Stud. Mar. Sci..

[25] Storelli M.M., Giachi L., Giungato D., Storelli A. (2011). Occurrence of heavy metals (Hg, Cd, and Pb) and polychlorinated biphenyls in salted anchovies. J. Food Prot..

[26] European Commission (2021). Analytical Quality Control and Method Validation Procedures for Pesticide Residues Analysis in Food and Feed (SANTE 11312/2021). https://www.eurl-pesticides.eu/userfiles/file/EurlALL/SANTE_11312_2021.pdf.

[27] European Union (2022). Commission Recommendation (EU) 2022/1431 of 24 August 2022 on the monitoring of perfluoroalkyl substances in food. OJEU.

[28] ISTAT. http://dati.istat.it/Index.aspx?QueryId=42869.

[29] EUMOFA (2018). European Market Observatory for Fisheries and Aquaculture Product: Anchovy Transformed in Italy. Case Study (February). https://www.eumofa.eu/documents/20178/111808/Price+structure+-+Anchovy+in+Italy.pdf.

[30] Mosconi G., Di Cesare F., Arioli F., Nobile M., Tedesco D.E., Chiesa L.M., Panseri S. (2022). Organohalogenated Substances and Polycyclic Aromatic Hydrocarbons in Fish from Mediterranean Sea and North Italian Lakes: Related Risk for the Italian Consumers. Foods.

[31] Chiesa L.M., Ceriani F., Caligara M., Di Candia D., Malandra R., Panseri S., Arioli F. (2018). Mussels and clams from the italian fish market. is there a human exposition risk to metals and arsenic?. Chemosphere.

[32] Søeborg T., Frederiksen H., Andersson A.M. (2012). Cumulative risk assessment of phthalate exposure of Danish children and adolescents using the hazard index approach. Int. J. Androl..

[33] EFSA CONTAM Panel (2012). EFSA Panel on Contaminants in the Food Chain (CONTAM); Scientific opinion on the risk for public health related to the presence of mercury and methylmercury in food. EFSA J..

[34] EFSA CONTAM Panel (2009). EFSA Panel on Contaminants in the Food Chain (CONTAM); Cadmium in food-Scientific opinion of the panel on contaminants in the food chain. EFSA J..

[35] EFSA CONTAM Panel (2010). EFSA Panel on Contaminants in the Food Chain (CONTAM); Scientific opinion on lead in food. EFSA J..

[36] EFSA CONTAM Panel (2014). EFSA Panel on Contaminants in the Food Chain (CONTAM); Scientific opinion on the risks to public health related to the presence of chromium in food and drinking water. EFSA J..

[37] EFSA CONTAM Panel (2009). EFSA Panel on Contaminants in the Food Chain (CONTAM); Scientific opinion on arsenic in food. EFSA J..

[38] EFSA (2021). Chronic dietary exposure to inorganic arsenic. EFSA J..

[39] Tokar E.J., Boyd W.A., Freedman J.H., Waalkes M.P., Klaassen C.D. (2013). Toxic effects of metals. Casarett and Doull’s Toxicology: The Basic Science of Poisons.

[40] EFSA (2004). Opinion of the Scientific Panel on contaminants in the food chain (CONTAM) to assess the health risks to consumers associated with exposure to organotins in foodstuffs. EFSA J..

[41] EFSA (2008). Safety of aluminium from dietary intake—Scientific Opinion of the Panel on Food Additives, Flavourings, Processing Aids and Food Contact Materials (AFC). EFSA J..

[42] EFSA (2015). Scientific opinion on the risks to public health related to the presence of nickel in food and drinking water. EFSA J..

